# Cholecystokinin induces crowing in chickens

**DOI:** 10.1038/s41598-019-40746-9

**Published:** 2019-03-08

**Authors:** Tsuyoshi Shimmura, Mai Tamura, Shosei Ohashi, Asuka Sasaki, Takamichi Yamanaka, Nobuhiro Nakao, Kunio Ihara, Shinsaku Okamura, Takashi Yoshimura

**Affiliations:** 1 Laboratory of Animal Integrative Physiology, Nagoya University, Nagoya, Aichi 464-8601 Japan; 2 Avian Bioscience Research Center, Graduate School of Bioagricultural Sciences, Nagoya University, Nagoya, Aichi 464-8601 Japan; 3 Center for Gene Research, Nagoya University, Nagoya, Aichi 464-8601 Japan; 4 Institute of Transformative Bio-Molecules (WPI-ITbM), Nagoya University, Nagoya, Aichi 464-8601 Japan; 5 Faculty of Applied Life Science, Nippon Veterinary and Life Science University, Musashino, Tokyo 180-8602 Japan; 6 Division of Seasonal Biology, National Institute for Basic Biology, Okazaki, Aichi 444-8585 Japan; 7 Department of Biological Production, Tokyo University of Agriculture and Technology, Fuchu, Tokyo 183-8509 Japan

## Abstract

Animals that communicate using sound are found throughout the animal kingdom. Interestingly, in contrast to human vocal learning, most animals can produce species-specific patterns of vocalization without learning them from their parents. This phenomenon is called innate vocalization. The underlying molecular basis of both vocal learning in humans and innate vocalization in animals remains unknown. The crowing of a rooster is also innately controlled, and the upstream center is thought to be localized in the nucleus intercollicularis (ICo) of the midbrain. Here, we show that the cholecystokinin B receptor (*CCKBR*) is a regulatory gene involved in inducing crowing in roosters. Crowing is known to be a testosterone (T)-dependent behavior, and it follows that roosters crow but not hens. Similarly, T-administration induces chicks to crow. By using RNA-sequencing to compare gene expression in the ICo between the two comparison groups that either crow or do not crow, we found that *CCKBR* expression was upregulated in T-containing groups. The expression of *CCKBR* and its ligand, cholecystokinin (*CCK*), a neurotransmitter, was observed in the ICo. We also showed that crowing was induced by intracerebroventricular administration of an agonist specific for CCKBR. Our findings therefore suggest that the CCK system induces innate vocalization in roosters.

## Introduction

There are many vertebrate and invertebrate animals that use sound for communication, and their patterns and functions are species-specific. For example, dogs bark to threaten other individuals^1^, and a mother cow moos to call her calf^2^. Interestingly, in contrast to human vocal learning, most animals can produce species-specific patterns of vocalization without learning them from their parents^3^, which is a phenomenon called innate vocalization. However, the underlying molecular basis of both vocal learning in humans and innate vocalization remains a mystery. Innate vocalization is a simpler behavior than human language and can therefore serve as an excellent model to uncover the molecular basis of vocalization.

The crowing of a rooster is most frequently observed before dawn^4^. Predawn crowing is a means for roosters to inform other individuals of their social status^5^. Rooster crowing is a form of innate vocalization, as a mature rooster fully develops the ability to crow even if his vocal learning is inhibited by either isolation or by surgical inhibition of his auditory senses from the time of hatching^6^. However, the molecules involved in regulating crowing have been not identified.

Steroid hormones such as T have multiple effects on morphology, physiology, and behavior. The social hierarchy of roosters is determined by certain cues such as comb size and crowing, and these are in turn affected by the concentration of T produced by the testes^7,8^. Roosters therefore crow but hens do not^7^. Although chicks do not crow, chick crowing can be induced by chronic T administration^9^. As these reports have shown, T regulates innate vocalization, but the genes that are regulated by T in this process remain unknown. In contrast with songbirds, which are capable of vocal learning, the intracerebral vocal pathway of chickens is much less complex^10^ (Supplementary Fig. S1). Although electrical stimulation of vocalization in the intercollicular nucleus (ICo) region in a number of bird species is controversial due to the complex organization of this area and the current applied^11–15^, the upstream center of vocalization has been proposed to be localized in the ICo of the midbrain^10^ (Supplementary Fig. S1). For example, bilateral lesions of the ICo result in muting^11^, and electrical stimulation produces calls that include crowing^16^. The androgen receptor (AR) is also localized to the ICo^17,18^. T binds to the androgen receptor and therefore is expected to regulate the expression of various downstream genes in the ICo. Therefore, these reports indicate that the T-dependent regulatory gene for crowing is expressed in the ICo of chickens.

In this study, we compared gene expression in the ICo using RNA-sequencing between the two comparison groups and showed that *CCKBR*, which encodes the cholecystokinin B receptor, is the gene that induces crowing.

## Results

### Crowing is a testosterone-dependent vocalization

**Figure 1:**
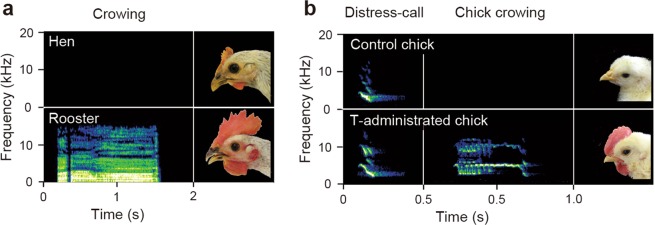
Crowing and comb size are dependent on testosterone. (**a**) In contrast with hens, roosters crow and have bigger combs. (**b**) While both control chicks and T-administered chicks emit distress calls, T-administered chicks also crow and have bigger combs (Fig. S2).

Mature roosters have bigger combs and can crow, while mature hens have smaller combs and do not crow (Fig. 1a). Chickens are social animals, and chicks emit a distress call when they are isolated from the group (Fig. 1b). However, as expected, when T is chronically administered to chicks through subcutaneous implantation of a silastic tube containing testosterone propionate, a prominent comb develops and the chicks begin to emit a crowing sound, which is clearly different from the sound of their distress calls (Fig. 1b, Supplementary Fig. S2a,b). For this study, we therefore generated two experimental groups that allow us to compare individuals that crow with individuals that do not. The first group included chicks and chicks administered T, while the second included hens and roosters.

### Genome-wide expression analysis to identify genes that induce crowing

Although the dorsomedial nucleus of the ICo (DM) controls sound production in song birds, the DM has not been characterized anatomically in chickens^3^ (Supplementary Fig. S1). Therefore, we first developed a method to punch out the ICo (Supplementary Fig. S3). We collected ICos from inbred chickens 2 h before light-onset when crowing was most frequently observed^4,5^. Next, we determined the precision of our punches of the ICo based by measuring the expression of *AR* using *in situ* hybridization (Supplementary Fig. S3), and obtained a total of 16 punches, consisting of four samples each from four groups (control chicks, T-administered chicks, hens, roosters). We went on to extract RNA from each ICo punch with which we prepared 16 cDNA libraries from each RNA extract, and performed RNA-sequencing using SOLiD 5500 (Thermo Fisher Scientific, MA). We amplified approximately fifty million reads per sample, aligned them against a chicken reference genome, counted the number of reads in each transcript, and performed statistical tests (*p* < 0.05, *false discovery rate* (*FDR*) < 0.1) and filtration (see Materials and Methods).

**Figure 2:**
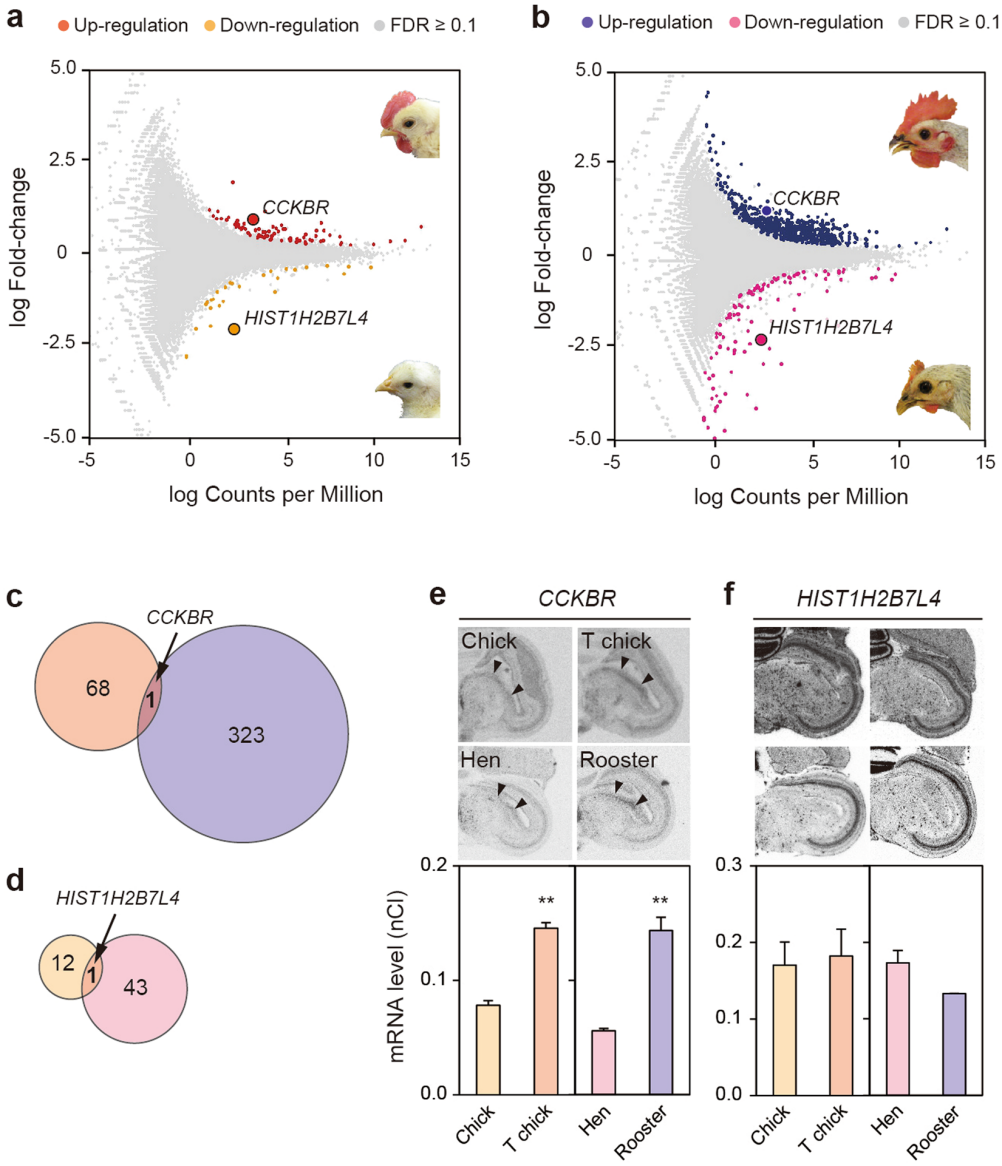
Identification of a gene that induces crowing using a functional genomics approach. (**a,b**) MA-plots of transcripts analyzed by RNA-sequencing. The number of differently expressed transcripts is indicated by colored dots, compared between T-administered chicks and control chicks (**a**) (82), and between roosters and hens (**b**) (368) (*p* < 0.05, *FDR* < 0.1). The photographs of chick heads are the same as Fig. S2 (**a**). (**c,d**) Combining the two comparisons, the upregulated *CCKBR* (**c**) and downregulated *HIST1H2B7L4* were identified as candidate genes (**d**). (**e,f**) *in situ* hybridization analysis showed that the gene expressions of *CCKBR* in the ICo (arrowhead) paralleled the upregulation observed with RNA-sequencing (*t* = 10.6 (control chicks versus T-administered chicks) and 6.8 (roosters versus hens), both *p* < 0.01, *t*-test, mean + SEM, *n* = 3–6, *e*), while ICo-specific expression was not observed for *HIST1H2B7L4* (*p* > 0.05, *n* = 3, *f*).

Comparison of control chicks with T-administered chicks identified 82 differently regulated transcripts (Fig. 2a), including 69 that were upregulated (Fig. 2c) and 13 that were downregulated (Fig. 2d). Additionally, we identified 368 transcripts (Fig. 2b), including 324 that were upregulated (Fig. 2c) and 44 that were downregulated (Fig. 2d) by comparing hens and roosters. To identify candidate genes whose expression was altered in both comparison groups, we combined the results from the two comparisons and identified two common genes, namely *CCKBR*, which was upregulated (Fig. 2c), and histone cluster 1, H2B-VII-like 4 (*HIST1H2B7L4*), which was downregulated (Fig. 2d). When we measured the expression level of these two genes in the ICo by using *in situ* hybridization, *CCKBR* showed differences that paralleled the upregulation observed with RNA-sequencing (Fig. 2e, *P* < 0.01, *n* = 3–6), while ICo-specific expression was not observed for *HIST1H2B7L4* (Fig. 2F, *P* > 0.05, *n* = 3). Therefore, we identified *CCKBR* as a candidate gene that regulates crowing.

### *CCKBR* induces crowing

**Figure 3:**
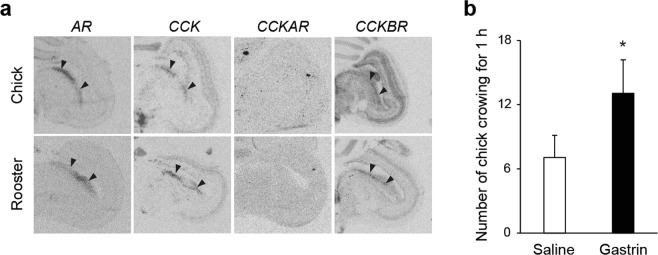
*CCKBR* induces chick crowing. (**a**) *AR*, *CCK*, and *CCKBR*, but not *CCKAR*, were expressed in the ICo (arrowhead). (**b**) Intracerebroventricular administration of gastrin, an agonist specific for *CCKBR*, induces chick crowing in a group situation (*t* = 2.2, *p* < 0.05, *t*-test, mean + SEM, *n* = 16).

The cholecystokinin system is activated by binding of the ligand cholecystokinin (*CCK*) to either cholecystokinin A receptor (*CCKAR*) or cholecystokinin B receptor (*CCKBR*). When we measured the expression level of these genes in the ICo by *in situ* hybridization, we detected the expression of *AR*, *CCK*, and *CCKBR* in the ICo, but not the expression of *CCKAR* (Fig. 3a). We therefore hypothesized that *CCK* binds to *CCKBR* in the ICo. We also determined if crowing is induced by intracerebroventricular administration of gastrin, an agonist specific for *CCKBR*. Since the ICo is in contact with the lateral ventricle (Supplementary Fig. S3), gastrin can reach the ICo if administered into the third ventricle. We also confirmed that chick crowing was observed when the chicks were placed in groups and tested this under the low-stress conditions (Supplementary Fig. S4). Under these conditions, we found that the chicks that received gastrin emitted chick crowing more frequently than the control chicks that received saline (Fig. 3b).

## Discussion

In this study, to identify genes that regulate innate vocalization in animals, we used rooster crowing as our model and performed functional genomics using RNA-sequencing. We compared individuals that crow with those that do not. To our surprise, despite the large behavioral difference, only a small number of genes showed large fold-change values (Fig. 2a,b). One possible explanation for this is that changes in gene expression are smaller in the specific nucleus of the brain compared with peripheral tissues^19^. However, through integrative functional genomics, including intracerebroventricular administration, we successfully identified *CCKBR* as a candidate gene that regulates crowing. *CCKAR* and *CCKBR* are ~50% identical in their sequences, and while *CCK* acts as a gastrointestinal hormone in peripheral tissues by binding to *CCKAR*, *CCK* also functions as a neurotransmitter in the brain by binding to *CCKBR*^20,21^. *CCKBR* also encodes a G-protein coupled receptor for CCK and gastrin, and has multiple effects on physiology and behavior through binding CCK and/or gastrin in the brain^20,21^. *CCKBR* expression was dependent on androgen (Fig. 2a,c), and *AR*, *CCK*, and *CCKBR* expression were observed in the ICo (Fig. 3a). It is therefore reasonable to speculate that androgens induce *CCKBR* expression in the ICo, accelerating the binding of CCK to CCKBR, which results in the induction of crowing.

CCK acts as a gastrointestinal hormone in peripheral tissues by binding to CCKAR^20,21^. In the brain, CCK instead functions as a neurotransmitter by binding to CCKBR^20,21^, which the results of our *in situ* hybridization agree with (Fig. 3a). The intracerebral CCK system involving CCKBR is activated by social stresses, such as when unfamiliar individual is suddenly introduced into home cage^22^. Crowing is also induced in roosters by social stressors such as when an unfamiliar individual crows (even if the crowing sound is played over a speaker). Induction is observed immediately, within tens of seconds when we presented social stimuli such as crowing by other individuals using a speaker^4,5^. Previous studies have also revealed that when mammals and chickens are subjected to intracerebroventricular administration of an agonist specific for CCKBR, stress-like anxiety behavior is observed immediately^23–26^. Therefore, it may be reasonable to conclude that activation of the CCK system in midbrain ICo induces crowing, which is indeed supported by the results that intracerebroventricular administration of an agonist specific for *CCKBR*-induced crowing (Fig. 3b).

In songbirds that show vocal learning, the innate and simple calls, but not learned songs, emerged by the electrical stimulus of the ICo^27–29^. Interestingly, the CCK system also exists in the ICo of songbirds^30,31^. Also, the ICo of birds corresponds to the midbrain periaqueductal gray (PAG) in mammals^3,10^. The innate vocalization of rodents is controlled by the PAG. Even in humans, involuntary vocalizations, such as when a person shrieks in response to an aversive stimulus, are regulated in PAG^3,10^. It was also revealed that CCK is a major transmitter in the PAG^32^. As these previous studies have indicated, the CCK system of vertebrates is highly conserved in the region of the brain involved in innate vocalization^33,34^. Therefore, the CCK system is of interest to understanding the evolution of innate vocalization in animals.

The molecular basis of innate vocalization had thus far not been determined. In this study, we showed that the involvement of the CCK system triggers the innate vocalization of animals. Most animals use species-specific innate vocalization for communications. Therefore, properly functioning innate vocalization by the CCK system may be critical for animal survival.

## Materials and Methods

### Animals

Inbred chickens of the GSP strain^35^ were used for RNA-seq and *in situ* hybridization where individual differences were expected to be small. Commercial White Leghorn chicks were used for administration experiments because many chicks can be used at the same time. The chickens were kept under a 12-h light:12-h dark cycle. The chickens had *ad libitum* access to water and feed. All operations were conducted under anesthesia using pentobarbital (25 mg/kg body weight). Brain samples were collected after euthanasia by decapitation. Animals were treated in accordance with the guidelines of Nagoya University. All experimental protocols were approved by Nagoya University.

### Sample preparation and collection

Chick gender of the GSP strain group was determined by PCR-based methods according to a previous report^36^. To induce chick crowing, male chicks received a hypodermic implantation with 35 mm silastic tubes (Dow Corning Toray, Japan) containing testosterone propionate (Internal diameter: 0.64 mm; External diameter: 1.19 mm; T-1875, Sigma-Aldrich, Japan) within 24 h of hatching^37^. Control male chicks were implanted with empty tubes. We confirmed that chick crowing in T-administered chicks was observed more frequently 4 d after T administration (Fig. S2). In the experiment to collect brain samples for RNA-seq, the crowing of the chicks was measured individually 3 d after hatching for 10 min in a single light- and sound-tight room using an IC recorder (ICD-UX300F, Sony, Tokyo Japan) connected to a microphone (ECM-CZ10, Sony). We visualized the sound using Sony Sound Forge Audio Studio ver. 9.0 (Sony), and counted the number of chick crowing sounds emitted according to a previous study^9^ (Figs 1A, S2). At day 5, we obtained brain samples 2 h before light-onset when rooster crowing was observed frequently^4,5^. Brains were rapidly removed, unilateral 2 mm-thick and 1 × 4 mm square punches of the ICo were collected (Fig. S2), and both the punches for RNA-seq and the other brain areas for validation by *in situ* hybridization were flash-frozen on dry ice.

For roosters and hens, we used chickens after they had reached 20 weeks of age at which the roosters of the GSP strain show complete crowing. Before brain sample collection, we confirmed that roosters did in fact crow through direct observation in the birds’ home cage after light onset. As with the chicks, we obtained brain samples 2 h before light-onset. Brains were removed, and 2 mm-thick and 1 × 6 mm square punches of the ICo were collected (Supplementary Fig. S2). We used only the punches for RNA-seq that passed validation by *in situ* hybridization (Supplementary Fig. S2).

### *In situ* hybridization

Frozen coronal sections (20 μm thick) of the ICo were examined with ^33^P-labelled oligonucleotide probes as previously described^38^. Densitometric analysis of hybridization signals was performed with Multi Gauge software (Fujifilm, Tokyo, Japan). Sections were counterstained by acetylcholinesterase staining. No hybridization signal was observed in the sense control. Gene-specific probes used are shown in Supplementary Table S1.

### RNA-sequencing and analysis

A total of 16 ICo punches consisting of four samples from each of the four groups (chicks, T-administered chicks, hens, and roosters) were used for RNA-sequencing. Ribosomal-RNA-minus RNAs (–rRNA) were prepared by removing DNA with the TURBO DNA-free Kit (Ambion) from isolated RNA before isolating ribosomal RNA with the RiboMinus Eukaryote Kit for RNA-Seq (Invitrogen). Next, we prepared the cDNA libraries from rRNAs using the SOLiD RNA-seq kit (Applied Biosystems). Quality of the RNA and cDNA obtained were confirmed with Agilent 2100 Bioanalyzer (Agilent Technologies). The cDNA libraries were sequenced by 5500 SOLiD (Life Technologies) with 75-bp and 35-bp paired-end reads at the Center for Gene Research, Nagoya University. The average (±SEM) number of reads generated are 50.8 ± 0.3 million per sample. RNA-seq reads were aligned to a chicken reference genome and annotation (Ensemble WASHUC2 (14 May 2012), iGenomes index and annotation packages) using Tophat (version 1.3.2)^39^ with the options for SOLiD color space. The Ensemble WASHUC2 was an early but current version when RNA-seq was performed. Aligned short reads were counted using Cufflinks (version 2.0.2)^39^. The normalization and differential analysis was performed using edge R^40^. Transcripts with inverted fold-change between the two experimental groups (e.g. up-regulation in chicks versus T-administered chicks, but down-regulation in roosters versus hens) and without annotation were filtered out.

### Intracerebroventricular administration

Chick gender of commercial White Leghorn chicks was determined by examining differences in feather development. Male chicks were selected and kept in a group as described above. Gastrin (Phoenix Pharmaceuticals, USA), an agonist specific for CCKBR and not CCKAR, was dissolved in saline together with 0.1% Evans Blue. Following a previous report^25^, chicks received gentle intracerebroventricular administration of 10 μl of 500 ng gastrin or saline at 5–8 days of age. The total amount of chick crowing amongst five chicks grouped together was counted for 1 h just after gastrin administration. Chick crowing was counted as described in the experiment involving T administration. After the experiments, we confirmed that the Evans Blue reached the lateral ventricles by sectioning the brains. We used the data of five chicks as a single set of data.

### Statistical analysis

Data were analyzed using Student’s *t*-test between the two groups. All data were analyzed by the statistical software program Statcel2.

## Supplementary information

Supplementary Information

## Data Availability

RNA-seq data are available from the NCBI Gene Expression Omnibus [GSE113699]. All other data are available from the authors upon request.
