# Supplementary material for: Cholecystokinin induces crowing in chickens
Source: Sci Rep. 2019 Mar 8;9:3978. doi: 10.1038/s41598-019-40746-9 (PMC6408447; doi:10.1038/s41598-019-40746-9)
Supplement: Supplementary file 1 — Supplementary Information [file 41598_2019_40746_MOESM1_ESM.pdf]

Supplemental Information

Cholecystokinin induces crowing in chickens

Tsuyoshi Shimmura, Mai Tamura, Shosei Ohashi, Asuka Sasaki, Takamichi Yamanaka, Nobuhiro Nakao, Kunio Ihara, Shinsaku Okamura, Takashi Yoshimura

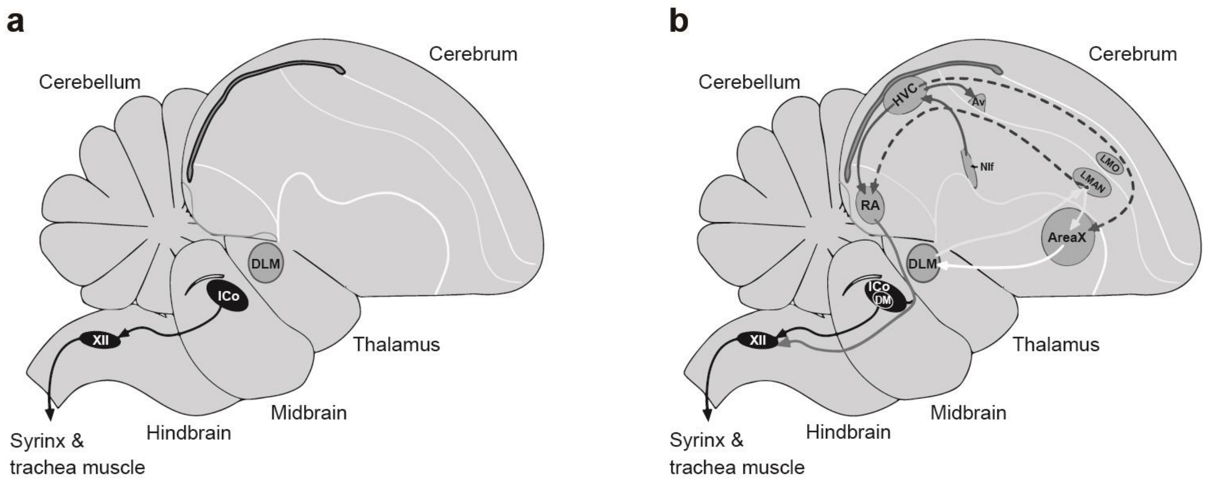

Supplemental Figure S1 | Schematic drawing of brain nucleus and connectivity of the vocal pathway in chicken (non-vocal learner) and zebra finch (vocal learner). (**a**) The forebrain seven song nucleus (**b**) is absent in chickens while the upstream center to produce sounds is located in the midbrain ICo. (**b**) Zebra finches have a complex vocal pathway, including the seven song nucleus. The schematic is adapted from Pfenning et al. (2014). Abbreviations: ICo, nucleus intercollicularis; XII, 12th motor nucleus; NIf, nucleus interfacialis; HVC, high vocal center; Av, avalanche; LMO, lateral oval nucleus of the mesopallium; LMAN, lateral magnocellular nucleus of the anterior nidopallium; RA, robust nucleus of the of arcopallium; DLM, dorsolateral nucleus of the medial thalamus; DM, dorsomedial nucleus of the ICo.

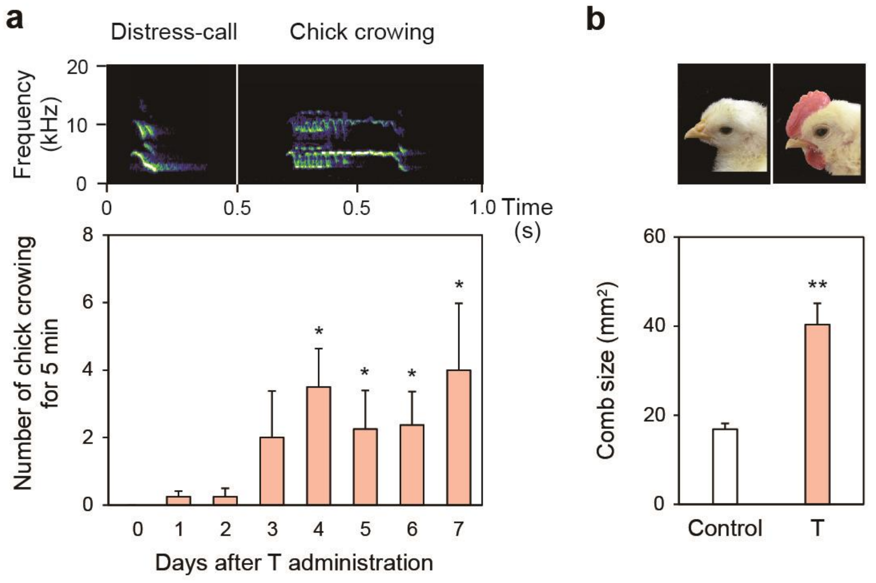

Supplemental Figure S2 | T-administration induces chick crowing. (**a**) Chick crowing of T-administered chicks was observed more frequently 4 d after T administration (H = 15.3, $p < 0.05$, Kruskal-Wallis' test; $p < 0.05$, Shirley-Williams' test, mean ± SEM, n = 8). (**b**) Comb size was bigger in T-administered chicks than control chicks at 7 d after T administration (t = -4.8, $p < 0.01$, t-test, mean + SEM, n = 8). This experiment was independent of the sample collection for RNA-seq (Figure 2).

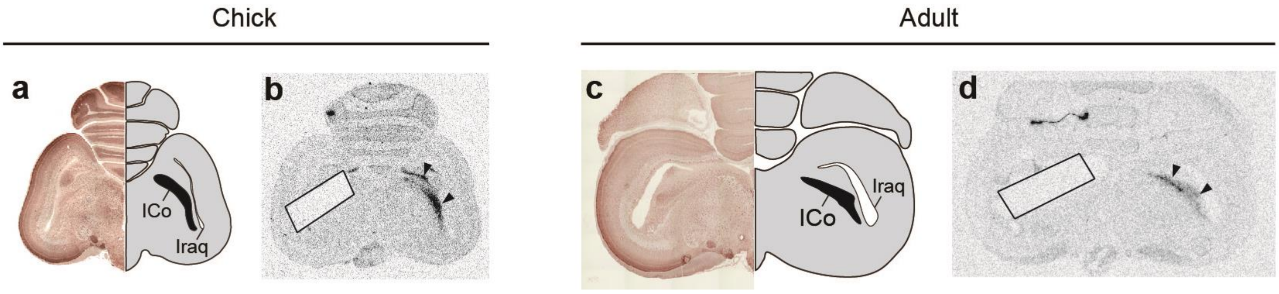

Supplemental Figure S3 | Validation of ICo punches by *in situ* hybridization. (**a, c**) Grey areas on schematic drawings indicate the ICo. On the opposite side are chromatic images that show the acetylcholine esterase stain. (**b, d**) Black arrowheads on the film indicate expression of the androgen receptor in the ICo. The white areas on the opposite side indicate that the ICo was punched out. Abbreviations: ICo, intercollicular nucleus; Iraq: lateral recess of the aqueduct.

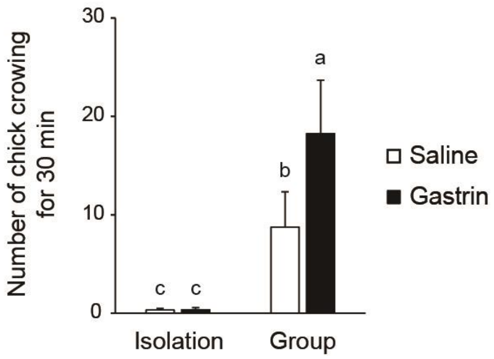

Supplemental Figure S4 | Chick crowing is observed more frequently when the chicks are kept in groups rather than when the chicks are isolated ($F_{1,62}$ = 54.1 [situation, $p < 0.01$], $F_{1,62}$ = 2.3 [treatment, $p > 0.01$], $F_{1,62}$ = 7.0 [interaction, $p < 0.05$], ANOVA; $^{a\text{-}c}p < 0.05$, Tukey-Kramer's test; mean + SE, n = 8–25). In group situations, 5 chicks were tested at the same time, and the total amount of chick crowing amongst the 5 chicks was counted and used as a single set of data.

Supplemental Table S1 | Probe sequences used in *in situ* hybridization.

Gene Symbol	Probe sequence (antisense sequence)
AR	5’- AAATAAAGGAGTGACCACCCCCAGGTGCTGCACACAGGGCGTTCC -3’<br>5’- GGAGATTATGCTCAGCTCTGGACAGGTCCTAGAAGCAGGAGAACC -3’<br>5’- GAGCTGCCGCATCCTGACGCATTGGCTGTACATTCTGGATTTGTG -3’<br>5’- TGCACAGCCCTGCTGCCTTCGCAGCAGCAAGTAGGTGTATGCAAG -3’
CCK	5’- ATGCTCTGTACCCTGTTCCCTAGGACAGAGAACCTCCCAGTGGAA -3'<br>5’- TCCATCCAGCCCATGTAGTCTCTGTCATTTATCCTGTGTGTGGGA -3'
CCKAR	5’- GCAGAAGAGGCAGAGCATTAAGTCACTGACTGCCAGGGACAGCAG -3'<br>5’- TTGTGCAGGTAGCACCCGTCTCCATCCTCGTATTTGGCGATGCTG -3'<br>5’- TCTTGGCCATCAGGTTGGCAGAAGAGCTGCTGCTTCTCACCCTGT -3'<br>5’- GTGTGTGTAAGAGCACTTGGAGAGCGAAGCCCTTGTCGTCTTCCC -3'
CCKBR	5'- GGTTGGGGATGAGGGTGAAGGGCATGCAGCAGAGCGCCAGCATCA -3'<br>5'- AATGAAGAAGAGGACGAGGAGCAGCAGGACATACCTGGAAGCGCA -3'<br>5'- AGCAGAGGAAGAACATGGCCACGATGACCACCAGCATGCGGATGA -3'<br>5'- CAGTGGTGTAGCTGAACTTGGAGAGCGATGCGTTGGCGTTGGCCA -3'
HIST1H2B7L4	5'- TGCACCTGCTTCAGCACCTTGTACACGTAGATCGAGTAGCTCTCC -3'<br>5'- GCACCTGCTTCAGCACCTTGTACACGTAGATCGAGTAGCTCTCCT -3'
